# Methionine Enkephalin Suppresses Osteocyte Apoptosis Induced by Compressive Force through Regulation of Nuclear Translocation of NFATc1

**DOI:** 10.1002/jbm4.10369

**Published:** 2020-05-26

**Authors:** Chisumi Sogi, Nobuo Takeshita, Wei Jiang, Siyoung Kim, Toshihiro Maeda, Michiko Yoshida, Toshihito Oyanagi, Arata Ito, Seiji Kimura, Daisuke Seki, Ikuko Takano, Yuichi Sakai, Ikuma Fujiwara, Shigeo Kure, Teruko Takano‐Yamamoto

**Affiliations:** ^1^ Department of Pediatrics, Graduate School of Medicine Tohoku University Sendai Japan; ^2^ Division of Orthodontics and Dentofacial Orthopedics Graduate School of Dentistry, Tohoku University Sendai Japan; ^3^ Young Dental Clinic Ulsan South Korea; ^4^ Minamihara Sakai Orthodontic Office Nagano Japan; ^5^ Department of Pediatrics Sendai City Hospital Sendai Japan; ^6^ Department of Biomaterials and Bioengineering Faculty of Dental Medicine, Hokkaido University Sapporo Japan

**Keywords:** APOPTOSIS, MECHANICAL STRESS, METHIONIN ENKEPHALIN, NFATc1, OSTEOCYTE

## Abstract

Mechanical stress stimulates bone remodeling, which occurs through bone formation and resorption, resulting in bone adaptation in response to the mechanical stress. Osteocytes perceive mechanical stress loaded to bones and promote bone remodeling through various cellular processes. Osteocyte apoptosis is considered a cellular process to induce bone resorption during mechanical stress‐induced bone remodeling, but the underlying molecular mechanisms are not fully understood. Recent studies have demonstrated that neuropeptides play crucial roles in bone metabolism. The neuropeptide, methionine enkephalin (MENK) regulates apoptosis positively and negatively depending on cell type, but the role of MENK in osteocyte apoptosis, followed by bone resorption, in response to mechanical stress is still unknown. Here, we examined the roles and mechanisms of MENK in osteocyte apoptosis induced by compressive force. We loaded compressive force to mouse parietal bones, resulting in a reduction of MENK expression in osteocytes. A neutralizing connective tissue growth factor (CTGF) antibody inhibited the compressive force‐induced reduction of MENK. An increase in osteocyte apoptosis in the compressive force‐loaded parietal bones was inhibited by MENK administration. Nuclear translocation of NFATc1 in osteocytes in the parietal bones was enhanced by compressive force. INCA‐6, which inhibits NFAT translocation into nuclei, suppressed the increase in osteocyte apoptosis in the compressive force‐loaded parietal bones. NFATc1‐overexpressing MLO‐Y4 cells showed increased expression of apoptosis‐related genes. MENK administration reduced the nuclear translocation of NFATc1 in osteocytes in the compressive force‐loaded parietal bones. Moreover, MENK suppressed Ca^2+^ influx and calcineurin and calmodulin expression, which are known to induce the nuclear translocation of NFAT in MLO‐Y4 cells. In summary, this study shows that osteocytes expressed MENK, whereas the MENK expression was suppressed by compressive force via CTGF signaling. MENK downregulated nuclear translocation of NFATc1 probably by suppressing Ca^2+^ signaling in osteocytes and consequently inhibiting compressive force‐induced osteocyte apoptosis, followed by bone resorption. © 2020 The Authors. *JBMR Plus* published by Wiley Periodicals, Inc. on behalf of American Society for Bone and Mineral Research.

## Introduction

Vertebrate skeletons are influenced by various kinds of mechanical stress during daily activities. Mechanical stress stimulates bone remodeling, which occurs through bone formation and resorption, resulting in bone adaptation to the mechanical stress.^(^ ^1^ ^)^ Osteocytes are the most abundant cells in bones and are embedded in bone matrices. Osteocytes have their dendritic processes through canaliculi to form an intercellular communication network via gap junctions.^(^ 2, 3 ^)^ Osteocytes perceive mechanical stress as mechanosensors, transform into biochemical signals, and regulate bone remodeling by transmitting signals related to bone formation and resorption.^(^ 4, 5 ^)^

Osteocytes express receptor activator of nuclear factor kappa B ligand (RANKL), an essential factor for osteoclastogenesis.^(^ ^6^ ^)^ Therefore, osteocytes are considered critical for osteoclastogenesis and bone resorption.^(^ 7, 8 ^)^ Importantly, not only living osteocytes, but osteocytes undergoing apoptosis also promote bone resorption.^(^ 9, 10, 11 ^)^ We previously reported that compressive force induces osteocyte apoptosis in the alveolar bones during experimental tooth movement in mice and in cell culture of chick calvaria‐derived osteocytes.^(^ 12, 13 ^)^ Our data suggest that osteocyte apoptosis occurs in response to compressive force and induces bone resorption. As to the mechanism of the compressive force‐induced osteocyte apoptosis, we revealed that connective tissue growth factor (CTGF) is a molecule that facilitates osteocyte apoptosis.^(^ ^13^ ^)^ However, the underlying molecular mechanisms of the compressive force‐induced osteocytes apoptosis are not fully understood.

Recent studies have demonstrated that neuropeptides play crucial roles in bone metabolism by regulating osteoblast and osteoclast function.^(^ 14, 15, 16 ^)^ The neuropeptide methionine enkephalin (MENK) is an endogenous opioid encoded by the proenkephalin (PENK) gene. It binds mainly to the delta opioid receptor (DOR) among three opioid receptors: mu, delta, and kappa.^(^ ^17^ ^)^ It is well known that MENK is expressed in nervous tissue, including the brain and spinal cord. MENK is also expressed in non‐nervous tissue, such as bone, heart, pancreas, and kidneys.^(^ 18, 19, 20, 21 ^)^ Its analgesic property is a major function of MENK; moreover, MENK has other functions, including anti‐inflammatory effects and regulation of embryonic organ development.^(^ 22, 23 ^)^ On the other hand, long‐term administration of opioids to alleviate cancer pain reduces bone mass, suggesting that opioids have a close relationship with bone remodeling.^(^ ^24^ ^)^ MENK is expressed in osteoblasts and mesenchymal stem cells, which are precursors of osteoblasts.^(^ 21, 25 ^)^ In addition, MENK inhibits osteoblast differentiation.^(^ ^26^ ^)^ These findings suggest the inhibitory effect of MENK on bone formation regulating osteoblast differentiation during bone remodeling. However, the role of MENK in bone resorption is still unknown.

McTavish and colleagues reported that PENK enhances apoptosis of human embryonic kidney (HEK) 293 cells and various human tumor cell lines.^(^ ^27^ ^)^ Conversely, activation of DOR suppresses apoptosis of neurons and hepatocytes.^(^ 28, 29 ^)^ These studies suggest that MENK regulates apoptosis positively and negatively depending on cell type. This notion led us to hypothesize that MENK has a novel role in bone resorption by regulating osteocyte apoptosis in response to compressive force. Thus, we first analyzed the expression of MENK in osteocytes and its effect on apoptosis using the mouse model of compressive force loading to cranial bones that we have already established.^(^ ^30^ ^)^ Furthermore, we analyzed the molecular mechanisms of MENK in osteocyte apoptosis induced by compressive force. Nuclear factor of activated T cells (NFAT) induces apoptosis in various types of cells, such as pulmonary artery smooth muscle cells, neurons, and glomerular mesangial cells.^(^ 31, 32, 33 ^)^ Lu and colleagues reported that NFATc2 activation by MENK significantly induces apoptosis of rat C6 glioma cells.^(^ ^34^ ^)^ Therefore, we focused on NFAT to elucidate the MENK‐mediated mechanism in osteocyte apoptosis.

## Material and Methods

### Application of compressive force to mouse parietal bones

Animal experiments were performed in accordance with the regulations for animal experiments and related activities at Tohoku University. All animal protocols were approved by the Institutional Animal Care and Use Committee of the Tohoku University Environmental and Safety Committee. Mice were housed in groups of up to four animals in a cage and kept at 20°C to 26°C with 12‐hour light/dark cycles. They had *ad libitum* access to fresh chow and water.

**Figure 1:**
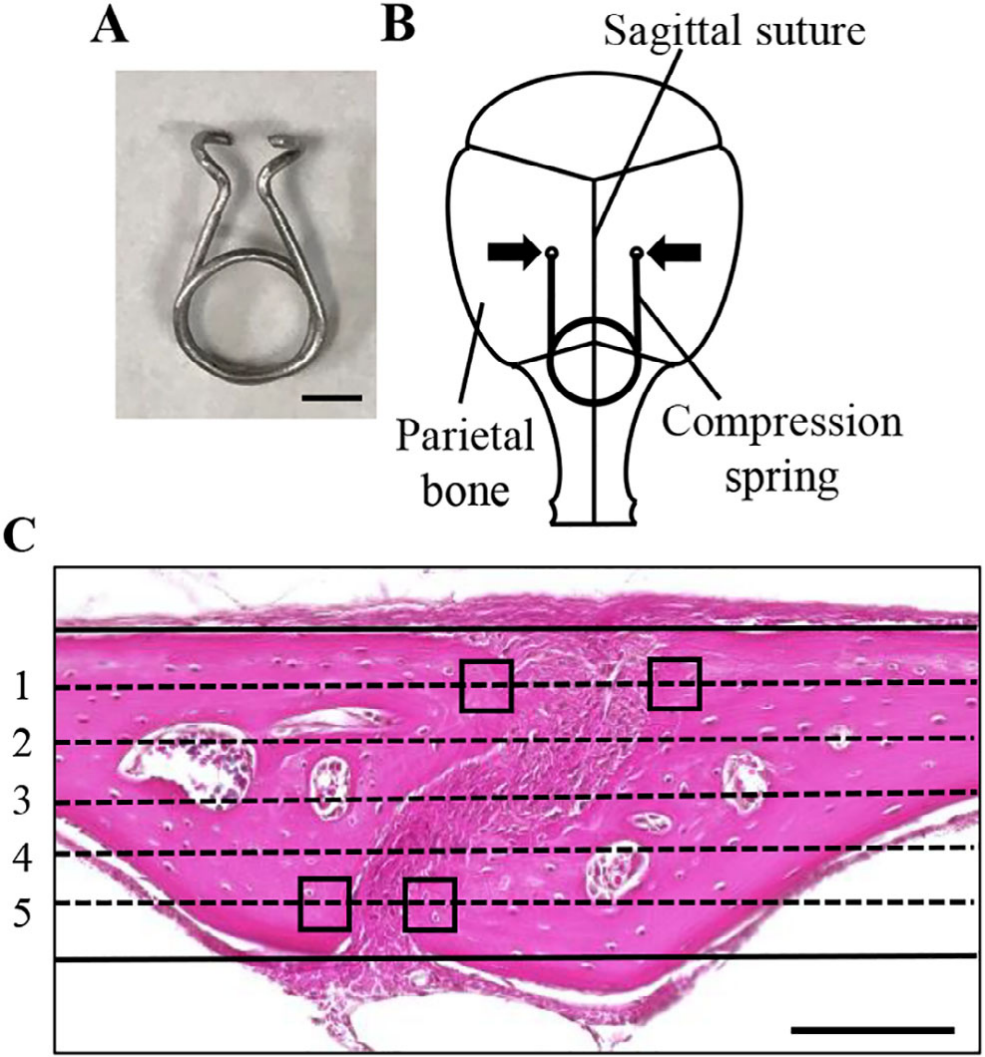
Application of compressive force to mouse parietal bones. (*A*) A spring for compressive force loading. Scale bar = 2 mm. (*B*) A schematic diagram of a compression spring placed on mouse parietal bones. To apply 0.2 N of compressive force to the parietal bones, a compression spring was set within holes drilled in the right and left parietal bones of 6‐week‐old mice. Arrows indicate the direction of compressive force on the parietal bones. (*C*) For histomorphometric analysis, five lines dividing the thickness of the parietal bones into six equal parts were drawn from dorsal to ventral sides on images of stained sections. The four square fields (30 × 30 μm) on the first and fifth lines, in contact with the edges of the right and left parietal bones adjacent to the sagittal suture, were set‐up in each specimen as the ROI. Scale bar = 100 μm.

Six‐week‐old male Institute of Cancer Research (ICR) mice (Clea Japan, Tokyo, Japan) were anesthetized by i.p. injection of medetomidine (0.3 mg/kg), midazolam (4 mg/kg), and butorphanol (5 mg/kg). A skin incision was made to expose the parietal bones. Two holes were made equidistant from a sagittal suture at the anteroposterior middle of the parietal bones using a round bur attached to a dental drill. The distance between the two holes was 5 mm. A spring that loads 0.2 N of compressive force onto the parietal bones was made by bending a 0.016‐inch diameter orthodontic beta–titanium wire (Fig. 1*A*). The spring was set within the holes in the parietal bones (Fig. 1 *B*), fully covered by skin, and the incision was closed by suturing. In the control group, identical surgical procedures were performed without spring installation.

Either 170 μL of 1‐mg/mL MENK (Sigma‐Aldrich, St. Louis, MO, USA), 100 μL of 25‐μM INCA‐6 (Cayman Chemical, Ann Arbor, MI, USA), or 100 μL of 10‐μg/mL neutralizing antihuman CTGF antibody (PeproTech, Rocky Hill, NJ, USA) were s.c. injected into the parietal bone area. For the negative control, the same volume of PBS or rabbit IgG (Sigma‐Aldrich) was injected. A compression spring was installed on parietal bones 2 hours after the MENK and INCA‐6 injection, or 6 hours after the CTGF antibody injection. Each injection was given again after surgery. During all of the experiments, mice were carefully monitored and no adverse events were observed.

### Histological analysis

After application of the 6‐hour compressive force, mice were perfusion fixed with 4% paraformaldehyde (PFA; Sigma‐Aldrich) under inhalation anesthesia with isoflurane. Calvariae, including the parietal bones, were dissected and immersed in 4% PFA at 4°C overnight. The specimens were decalcified with 20% ethylene‐diaminetetraacetic acid (pH 7.4) at 4°C for 7 days. The specimens were then embedded in paraffin, sectioned at 6 μm, and stained with hematoxylin (Sigma‐Aldrich) and eosin (Wako, Osaka, Japan).

### Immunohistochemical analysis

Deparaffinized sections were immersed in 0.01 M citrate solution and microwaved for 2 min, then incubated at room temperature (RT) for 30 min. The sections were treated with 10% donkey serum (Sigma‐Aldrich) in PBS at RT for 1 hour and incubated with primary antibodies against MENK (1:40; ImmunoStar, New Richmond, WI, USA), DOR (1:100; Abcam, Cambridge, UK), NFATc1 (1:40, Abcam), or NFATc3 (1:50; ProteinTech, Chicago, IL, USA) at RT for 2 hours or at 4°C overnight. In the negative controls, the primary antibodies were replaced by rabbit IgG (Sigma‐Aldrich). After washing with 0.3% TritonX‐100 (Nacalai Tespue, Kyoto, Japan) in PBS, the sections were incubated with donkey antirabbit‐IgG Alexa Fluor 568 (1:500; Invitrogen, Carlsbad, CA, USA) at RT for 1 hour. 4,6‐diamidino‐2‐phenylindole (DAPI; 1:1000; SeraCare, Milford, MA, USA) was used for nuclei detection. Fluorescent signals were visualized using a confocal laser scanning microscope system (C2si; Nikon, Tokyo, Japan). To quantify the fluorescence‐positive osteocytes, the fluorescent images were analyzed using ImageJ software (NIH, Bethesda, MD, USA; https://imagej.nih.gov/ij/); thresholds of 40 and 255 were used in red channel images. We determined the thresholds by superimposing binary images on the corresponding fluorescent images and confirming overlaps of the signals on the binary images and on the fluorescent images, according to our previous report.^(^ ^35^ ^)^ In our analysis of nuclear translocation of NFAT, fluorescent NFAT signals that contained nuclei of osteocytes were considered positive.

For the detection of active caspase‐3, deparaffinized sections were treated with 3% H_2_O_2_ in methanol and incubated overnight at 4°C with rabbit antihuman cleaved caspase‐3 (1:100; Cell Signaling Technology, Danvers, MA, USA) in Can Get Signal Immunostain Solution B (Toyobo, Osaka, Japan). After incubation with a peroxidase‐conjugated secondary antibody (Histofine Simple Stain Mouse MAX‐PO; Nichirei Bioscience, Tokyo, Japan) at RT for 30 min, the signals were visualized with 3,3‐diaminobenzi‐dine tetrahydrochloride (DAB; Nichirei, Tokyo, Japan). The images obtained from caspase‐3 immunohistochemistry were analyzed using ImageJ (NIH); thresholds of 0 and 200 were used to quantify the caspase‐3‐positive osteocytes.

### Terminal deoxynucleotidyl transferase‐mediated dUTP nick end labeling (TUNEL) staining

Apoptotic cell death was detected using the Apoptosis in situ Detection Kit (Wako Laboratories, Osaka, Japan) according to the manufacturer's protocol. Deparaffinized sections were incubated in prewarmed protease solution at 37°C for 10 min. For labeling 3′terminals of DNA, TdT reaction solution was pipetted onto the sections and incubated at RT for 2 hours. Endogenous peroxidase was inactivated using 3% H_2_O_2_ for 5 min. Peroxidase‐conjugated antibody solution was pipetted onto the sections at 37°C for 10 min. Apoptotic nuclei were then visualized by DAB (Nichirei) at RT for 8 min. Hematoxylin was used for counterstaining. The images obtained from TUNEL staining were analyzed using ImageJ imaging software (NIH). Thresholds of 0 and 160 were used to quantify the TUNEL‐positive osteocytes. TUNEL signals overlapping with nuclei of osteocytes were considered positive.

### Histomorphometric analysis

Five lines, which divide the thickness of the parietal bones adjacent to the sagittal suture into six equal parts, were drawn from the dorsal to the ventral side on images of stained sections. The four square fields (30 × 30 μm) on the first and fifth lines, in contact with edges of the right and left parietal bones adjacent to the sagittal suture, were set‐up as the ROI to quantitatively evaluate TUNEL‐positive osteocytes, expression of MENK, DOR, and caspase‐3 in osteocytes, and nuclear translocation of NFATc1 and NFATc3 in osteocytes (Fig. 1*C*). The number of osteocytes and the number of TUNEL‐ or immunohistochemically positive osteocytes in four ROIs in a section were used for calculation of ratio of positive osteocytes.

### Cell culture

MLO‐Y4 cells were cultured in α modified essential medium (Wako) supplemented with 5% fetal bovine serum (FBS; GE Healthcare, Buckinghamshire, UK), 5% bovine serum (Thermo Fisher Scientific, Waltham, MA, USA), 100‐U/mL penicillin, and 100‐μg/mL streptomycin (Thermo Fisher Scientific) on culture plates coated with 0.3‐mg/mL type I collagen (Nitta Gelatin, Osaka, Japan). For a MENK administration experiment, cells were seeded onto coated 12‐well plates at 1.4 × 10^4^ cells/cm^2^, and MENK was administered after 24 hours. Total mRNA was isolated from cells after 48 hours.

To load compressive force onto MLO‐Y4 cells, the cells were seeded at a density of 1.4 × 10^4^ cells/cm^2^ on silicone chambers (size 32 × 32 mm; Strex, Osaka, Japan) coated with 0.01% poly‐D‐lysine (Trevigen, Gaithersburg, MD, USA), and 0.3‐mg/mL type I collagen and cultured for 2 days. Compressive force was generated by 18% constriction of the chambers using a Strex cell stretch system (Strex); it was loaded onto MLO‐Y4 cells for 6 hours. In the control groups, MLO‐Y4 cells were cultured on the stretch chambers without compressive force loading. Cells were harvested to isolate total RNA using RNeasy Kit (Qiagen, Venlo, The Netherlands).

### Construction of lentiviral vector

The mouse NFATc1 open‐reading frame was amplified by PCR and cloned into the XhoI and XbaI sites of the pLVSIN‐IRES‐ZsGreen1 lentiviral vector (Takara Bio, Shiga, Japan). Viral particles were produced in 293 T Lenti‐X cell line (Clontech Laboratories, Palo Alto, CA, USA) using Lentiviral High Titer Packaging Mix (Takara Bio) following the manufacturer's instructions, and titered using Lenti‐X qRT‐PCR Titration Kit (Clontech).

### RNA isolation from parietal bones

RNA isolation from the mouse parietal bones was performed according to our previous report.^(^ ^36^ ^)^ Mice were sacrificed by cervical dislocation; the parietal bones were quickly dissected. After removing the periosteum and the dura mater, 0.5 mm of parietal bones on either side were isolated and minced with scissors. The specimens were immersed in TRIzol Reagent (Thermo Fisher Scientific) and centrifuged. Total mRNA was then isolated using the RNeasy Kit.

### Real‐time PCR

cDNA was synthesized from 0.4 μg of total mRNA using a PrimeScript RT Reagent Kit (Takara Bio). Real‐time PCR was performed using a Thermal Cycler Dice Real Time System (Takara Bio). The reaction volume was 25 μL, which contained 2 μL of cDNA, 12.5 μL of TB Green Premix Ex Taq II (Takara Bio), and 0.4 μM of sense and antisense primers. The primer sequences are listed in Table 1. The reactions consisted of 40 cycles of 5 s at 95°C and 30 s at 60°C. Relative expression of mRNA was normalized to that of hypoxanthine‐guanine phosphoribosyltransferase (HPRT) and analyzed by the ΔΔCT method.

**Table 1:** Primer for Real‐Time PCR

Gene name	Forward primer (5′‐3′)	Reverse primer (3′‐5′)
PENK	ATGCAGCTACCGCCTGGTTC	CTTGGCTAGCAAGTGGCTCTCA
DOR	CAGACAGCAGACTCCTGAAGCA	ACCGGCTGGGGTCAGCTCTAA
NFATc1	TGGAGATGGAAGCAAAGACTG	GCAGACATAGAAACTGACTTGGACG
NFATc2	AGGCTTTAGATGGACTGGGTGC	TGATGTCTGGGATGCTCTCACAG
NFATc3	GAGTGCCTATAAGTGAGACAC	GGATCCAACTACCTAGAGTAAC
NFATc4	CCCTACCAGACTCATCTCT	TAAGGAGTCTCATAAGCAGG
Caspase‐3	CTGCCGGAGTCTGACTGGAA	ATCAGTCCCACTGTCTGTCTCAATG
Caspase‐8	CAGGAGGAACAAGGGAAAGCAGTG	GAGAGCACACATCATTAGGGAG
Caspase‐9	TCCTGCCTCTTATTCCCAAGTTC	TGTATGCTCTGTGCTCACCTGG
Calmodulin	GTCTGTAACCTCTTGGTGAT	TACCCAGCTTCTACAGACTT
Calcineurin A	GTTACGGGTTACTACTGGTT	CTTGAGGCTGTAGTGAATG
Calcineurin B	AACCCTTCAGATGTCTAGTG	GGTAATCAGTACAGGTCAGTC
HPRT	AGGCAGATGGCCACAGGACTA	TTGTTGTTGGATATGCCCTTGACTA

DOR = delta opioid receptor; HPRT = hypoxanthine‐guanine phosphoribosyltransferase; PENK = proenkephalin.

### Western blot analysis

Whole‐cell proteins from MLO‐Y4 cells were prepared using Cell Lytic M Lysis Reagent (Sigma‐Aldrich) containing Protease Inhibitor Cocktail (Sigma‐Aldrich). The protein samples were boiled in sodium dodecyl sulfate (SDS) sample buffer, separated by SDS polyacrylamide gel electrophoresis, then transferred to polyvinylidene fluoride membranes. The membranes were treated with blocking buffer and incubated overnight at 4°C with rabbit antihuman cleaved caspase‐3 (1:500; Cell Signaling Technology) or mouse antihuman glyceraldehyde‐3‐phosphate dehydrogenase (GAPDH; 1:5000; ProteinTech) antibodies. The membranes were then incubated with horseradish peroxidase‐conjugated secondary antibodies, and developed with a chemiluminescence detection reagent. Chemiluminescent signals were acquired using the Fusion FX Imaging System (Vilber Lourmat, Marne La Vallée, France).

### Intracellular Ca^2+^ measurement

MLO‐Y4 cells were cultured on coated 3.5‐cm glass bottom dishes for 24 hours, and MENK was administered into the culture for an additional 24 hours. The cells were loaded with recording buffer (200‐mM HEPES, 115‐mM NaCl, 5.4‐mM KCl, 0.8‐mM MgCl_2_, 1.8‐mM CaCl_2_, 13.8‐mM glucose, and pH 7.4) containing 5‐μM Fura‐2 AM (Thermo Fisher Scientific), 1.25‐mM probenecid (Thermo Fisher Scientific), and 0.04% Pluronic F‐127 (Thermo Fisher Scientific) for 30 min at 37°C. Fluorescent images were captured with an Olympus IX71 (Olympus, Tokyo, Japan) and Hamamatsu EM‐CCD digital camera (Hamamatsu Photonics, Shizuoka, Japan) at the rate of 1 frame every 5 s for 5 min. One min after image capturing, 100‐nM ionomycin (Wako) was added to the recording buffer. The ratio of fluorescence at wavelengths of 340 and 380 nm were measured in 10 randomly selected cells in each dish, and the average was considered the fluorescence intensity of the sample. The fluorescence intensity in the cells just before ionomycin treatment was regarded as 1. The relative increase in intracellular calcium was assessed using Meta Fluor Software (Molecular Devices, Tokyo, Japan).

### Statistical analysis

All data are presented as mean ± SD. Data were statistically analyzed by Student's *t* test to compare differences between two groups. For more than two groups, we used an ANOVA, followed by the Tukey–Kramer test. *p* Values less than 0.05 were considered statistically significant.

## Results

### Compressive force reduced expression of MENK in osteocytes

**Figure 2:**
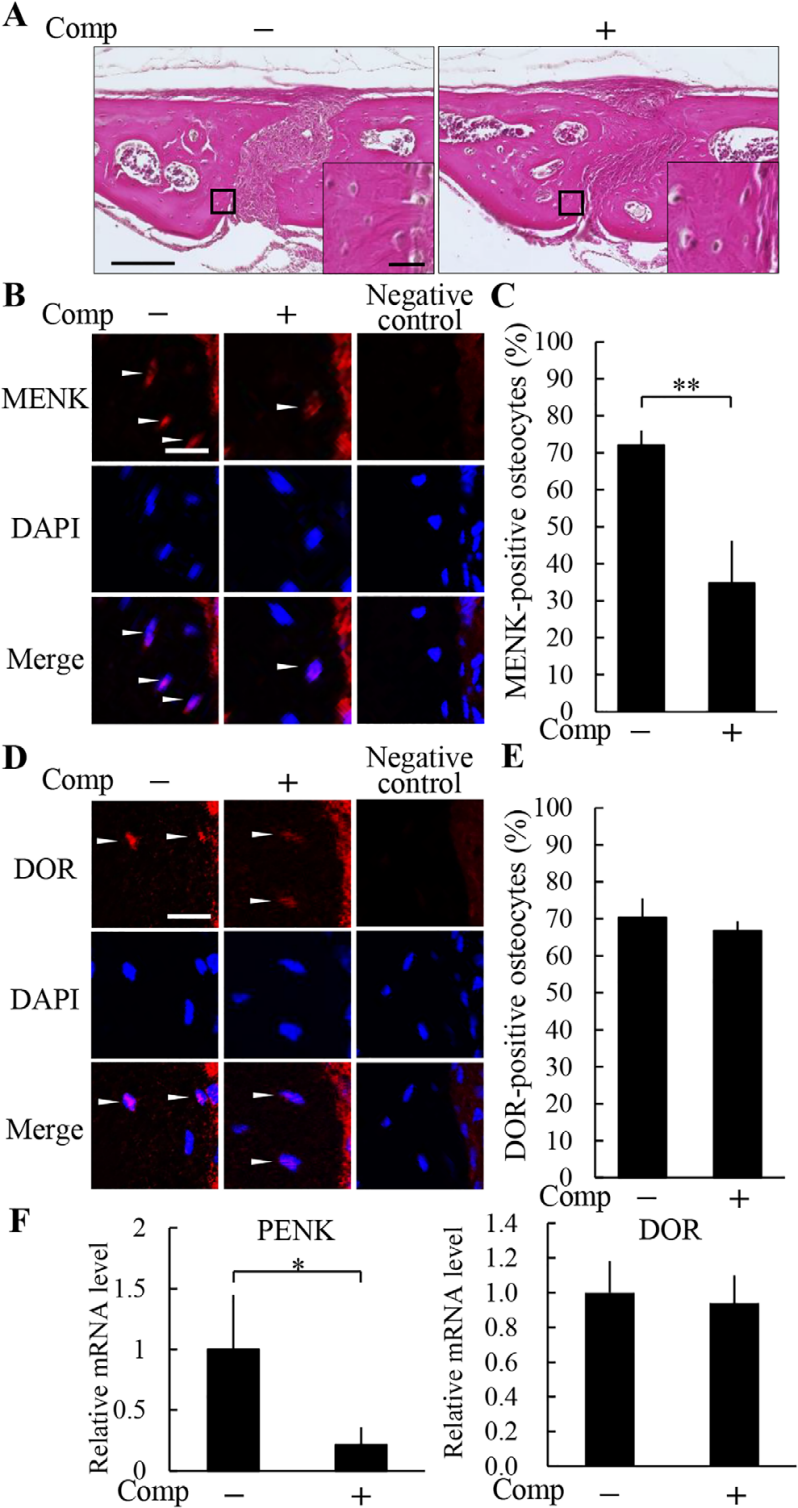
Compressive force reduced expression of methionine enkephalin (MENK) in osteocytes. (*A*) Histological analysis of the parietal bones at 6 hours after the application of compressive force was performed by H&E staining. A magnified picture of the ROI is shown in the lower right corner of each picture. Scale bar = 100 μm, and 10 μm in insets. The expression of MENK (*B,C*) and the delta opioid receptor (DOR) (*D,E*) in the parietal bones was analyzed by immunohistochemistry (*B,D*), and the ratio of positive osteocytes (C,E) was calculated (four animals per group). Arrowheads indicate immunohistochemically positive osteocytes. Scale bar = 10 μm. Comp = compressive force loading. **P < 0.01. (*F*) Compressive force was loaded onto MLO‐Y4 cells for 6 hours and expression of proenkephalin (PENK) and DOR was analyzed by real‐time PCR. Comp = Compressive force loading. *P < 0.05; *n* = 3.

Compressive force of 0.2 N loading onto the mouse parietal bones for 6 hours narrowed the suture width between the edges of the right and left parietal bones adjacent to the sagittal suture (Fig. 2*A*). Histological analysis showed no obvious morphological change of parietal bones and signs of an acute inflammatory response, such as neutrophil infiltration or edema (Fig. 2*A*). We analyzed expression of MENK and its main receptor DOR in osteocytes by immunofluorescence staining. MENK was expressed in 72.1 ± 3.9% of osteocytes in the nonloaded group, whereas the positive osteocyte ratio was significantly decreased to 34.7 ± 11.5% in the loaded group (Fig. 2*B*,*C*). The number of MENK‐positive osteocytes and the total number of osteocytes were 7.0 ± 1.7 and 9.8 ± 2.5, and 3.0 ± 1.4 and 8.5 ± 1.7 in the nonloaded and loaded groups, respectively. In contrast, there was no significant difference in the expression of DOR between the nonloaded and the loaded groups (Fig. 2*D*,*E*). The number of DOR‐positive osteocytes and the total number of osteocytes were 7.0 ± 0.8 and 10.0 ± 1.4, and 7.5 ± 0.6 and 11.3 ± 1.0 in the nonloaded and loaded groups, respectively. The negative controls of MENK and DOR immunohistochemistries showed no apparent immunopositivity in osteocytes (Fig. 2*B*,*D*). Consistent with in vivo data, compressive force downregulated expression of PENK mRNA, which encodes MENK, but did not significantly affect DOR mRNA expression in MLO‐Y4 cells (Fig. 2*F*).

### A neutralizing CTGF antibody inhibited the decrease in MENK expression in the compressive force‐loaded osteocytes

**Figure 3:**
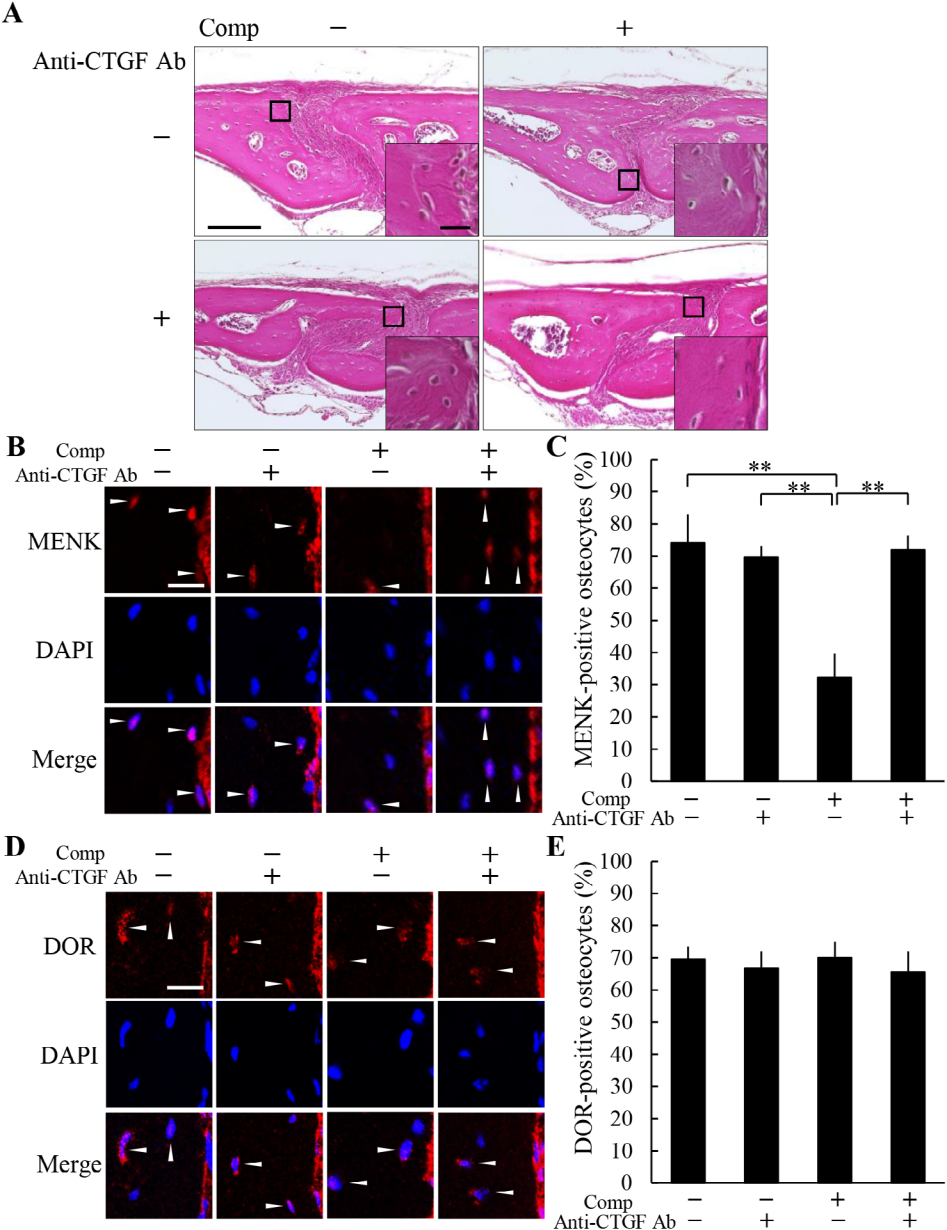
A neutralizing connective tissue growth factor (CTGF) antibody inhibited the decrease in methionine enkephalin (MENK) expression in the compressive force‐loaded osteocytes. (*A*) Histological analysis of the parietal bones at 6 hours after application of compressive force was performed by H&E staining. A neutralizing CTGF antibody was injected s.c. into the parietal bone area prior to the application of compressive force. A magnified picture of the ROI is shown in the lower right corner of each picture. Scale bar = 100 μm, and 10 μm in insets. The expression of MENK (*B,C*) and the delta opioid receptor (DOR) (D,E) in the parietal bones of each group was analyzed by immunohistochemistry (*B,D*), and the ratio of positive osteocytes (*C,E*) was calculated (four animals per group). Arrowheads indicate immunohistochemically positive osteocytes. Scale bar = 10 μm. Comp = compressive force loading, anti‐CTGF Ab = neutralizing CTGF antibody. **P < 0.01.

We previously reported that expression of CTGF in osteocytes is upregulated by a compressive force of 0.2 N loading for 6 hours in the same compressive force‐loaded mouse model as in the present study.^(^ ^13^ ^)^ To examine whether the increased CTGF influences the expression of MENK and DOR in the compressive force‐loaded osteocytes, we applied a neutralizing CTGF antibody to the parietal bones prior to compressive force loading. No obvious histological change was observed by administration of the neutralizing CTGF antibody in both the nonloaded and the loaded groups (Fig. 3*A*). In the nonloaded groups, MENK‐positive osteocyte ratios without and with the neutralizing CTGF antibody were 74.1 ± 8.9% and 69.7 ± 3.5%, respectively; there was no significant difference between these groups (Fig. 3*B*,*C*). In the loaded groups, MENK‐positive osteocyte ratio without the neutralizing CTGF antibody was 32.2 ± 7.4%; it was significantly lower than the nonloaded groups (Fig. 3*B*,*C*). The ratio in the loaded group with the neutralizing CTGF antibody was 71.8 ± 4.6%; it was significantly higher than the loaded group without the neutralizing CTGF antibody and was comparable to the nonloaded groups (Fig. 3*B*,*C*). The number of MENK‐positive osteocytes and the total number of osteocytes were 8.8 ± 1.5 and 12.0 ± 2.9, and 7.5 ± 1.0 and 10.8 ± 1.3 in the nonloaded groups without and with the neutralizing CTGF antibody, respectively, and 2.8 ± 1.0 and 8.5 ± 1.7, and 6.3 ± 1.5 and 8.8 ± 2.4 in the loaded groups without and with the neutralizing CTGF antibody, respectively. DOR‐positive osteocyte ratios in the nonloaded groups without and with the neutralizing CTGF antibody were 69.6 ± 3.9% and 66.8 ± 5.1%, respectively (Fig. 3*D*,*E*). The ratios in the loaded groups without and with the neutralizing CTGF antibody were 70.1 ± 4.8% and 65.4 ± 6.5%, respectively (Fig. 3*D*,*E*). There was no significant difference in DOR expression between any of the groups. The number of DOR‐positive osteocytes and the total number of osteocytes were 6.8 ± 1.0 and 9.8 ± 1.7, and 8.0 ± 0.8 and 12.0 ± 1.2 in the nonloaded groups without and with the neutralizing CTGF antibody, respectively, and 8.3 ± 1.3 and 11.8 ± 1.3, and 7.3 ± 1.0 and 11.3 ± 2.4 in the loaded groups without and with the neutralizing CTGF antibody, respectively.

### MENK suppressed osteocyte apoptosis induced by compressive force loading

**Figure 4:**
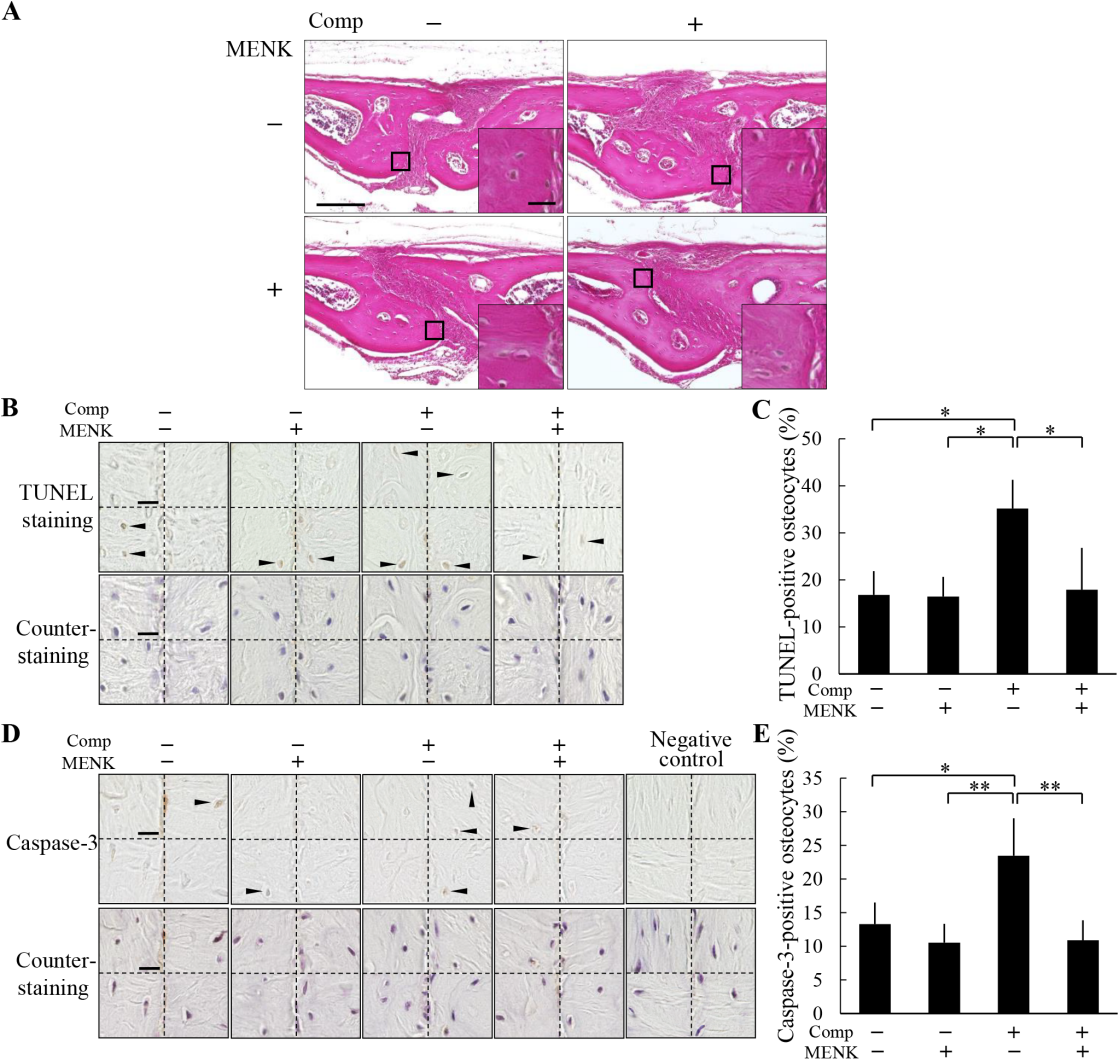
Methionine enkephalin (MENK) suppressed osteocyte apoptosis induced by compression force loading. (*A*) Histological analysis of the parietal bones at 6 hours after application of compressive force was performed by H&E staining. MENK was injected s.c. into the parietal bone area prior to application of compressive force. A magnified picture of the ROI is shown in the lower right corner of each picture. Scale bar = 100 μm, and 10 μm in insets. (*B*) TUNEL staining and subsequent counterstaining with hematoxylin of the parietal bones were performed. All four ROIs in a section in each group are shown. Arrowheads indicate TUNEL‐positive osteocytes. Scale bar = 10 μm. (*C*) TUNEL‐positive ratio of osteocytes was calculated (four animals per group). (*D*) Caspase‐3 immunohistochemistry and subsequent counterstaining with hematoxylin of the parietal bones were performed. All four ROIs in a section in each group are shown. Arrowheads indicate caspase‐3‐positive osteocytes. Scale bar = 10 μm. (*E*) Caspase‐3‐positive ratio of osteocytes was calculated (four animals per group). Comp = compressive force loading. *P < 0.05, **P < 0.01.

Our previous study showed that the increased CTGF by compressive force loading induces osteocyte apoptosis in mouse parietal bones.^(^ ^13^ ^)^ To evaluate the effect of MENK on the osteocyte apoptosis induced by compressive force, exogenous MENK was s.c. injected into the parietal bone area prior to compressive force loading. There was no obvious histological change caused by MENK administration in the nonloaded and the loaded groups (Fig. 4*A*). TUNEL‐positive osteocyte ratios without and with MENK in the nonloaded groups were 16.8 ± 5.1% and 16.4 ± 4.2%, respectively (Fig. 4*B*,*C*). In the loaded group without MENK, TUNEL‐positive osteocyte ratio significantly increased to 35.2 ± 6.2% compared with the nonloaded groups (Fig. 4*B*,*C*). On the other hand, the MENK administration in the loaded group significantly decreased TUNEL‐positive osteocyte ratio to 17.9 ± 8.9% compared with the loaded group without MENK, resulting in the comparable TUNEL‐positive osteocyte ratio to the nonloaded groups (Fig. 4*B*,*C*). The number of TUNEL‐positive osteocytes and the total number of osteocytes were 1.5 ± 0.6 and 8.8 ± 1.0, and 1.5 ± 0.6 and 9.0 ± 2.2 in the nonloaded groups without and with MENK, respectively, and 3.8 ± 0.5 and 10.8 ± 1.0, and 1.8 ± 1.0 and 9.8 ± 1.9 in the loaded groups without and with MENK, respectively. We also examined expression of active caspase‐3 in osteocytes. In the nonloaded groups, caspase‐3‐positive osteocyte ratios without and with MENK were 13.3 ± 3.2% and 10.5 ± 2.8%, respectively (Fig. 4*D*,*E*). In the loaded groups, caspase‐3‐positive osteocyte ratio without MENK was 23.5 ± 5.6%; it was significantly higher than the nonloaded groups (Fig. 4*D*,*E*). The ratio in the loaded group with MENK was 10.9 ± 3.0%; it was significantly lower than the loaded group without MENK and comparable to the nonloaded groups (Fig. 4*D*,*E*). The number of caspase‐3‐positive osteocytes and the total number of osteocytes were 1.5 ± 0.6 and 11.0 ± 1.9, and 1.3 ± 0.5 and 11.8 ± 2.1 in the nonloaded groups without and with MENK, respectively, and 2.3 ± 0.5 and 9.8 ± 1.7, and 1.3 ± 0.5 and 11.3 ± 1.3 in the loaded groups without and with MENK, respectively. The negative control of caspase‐3 immunohistochemistry showed no apparent immunopositivity in osteocytes (Fig. 4*D*).

### Compressive force induced nuclear translocation of NFATc1 in osteocytes

**Figure 5:**
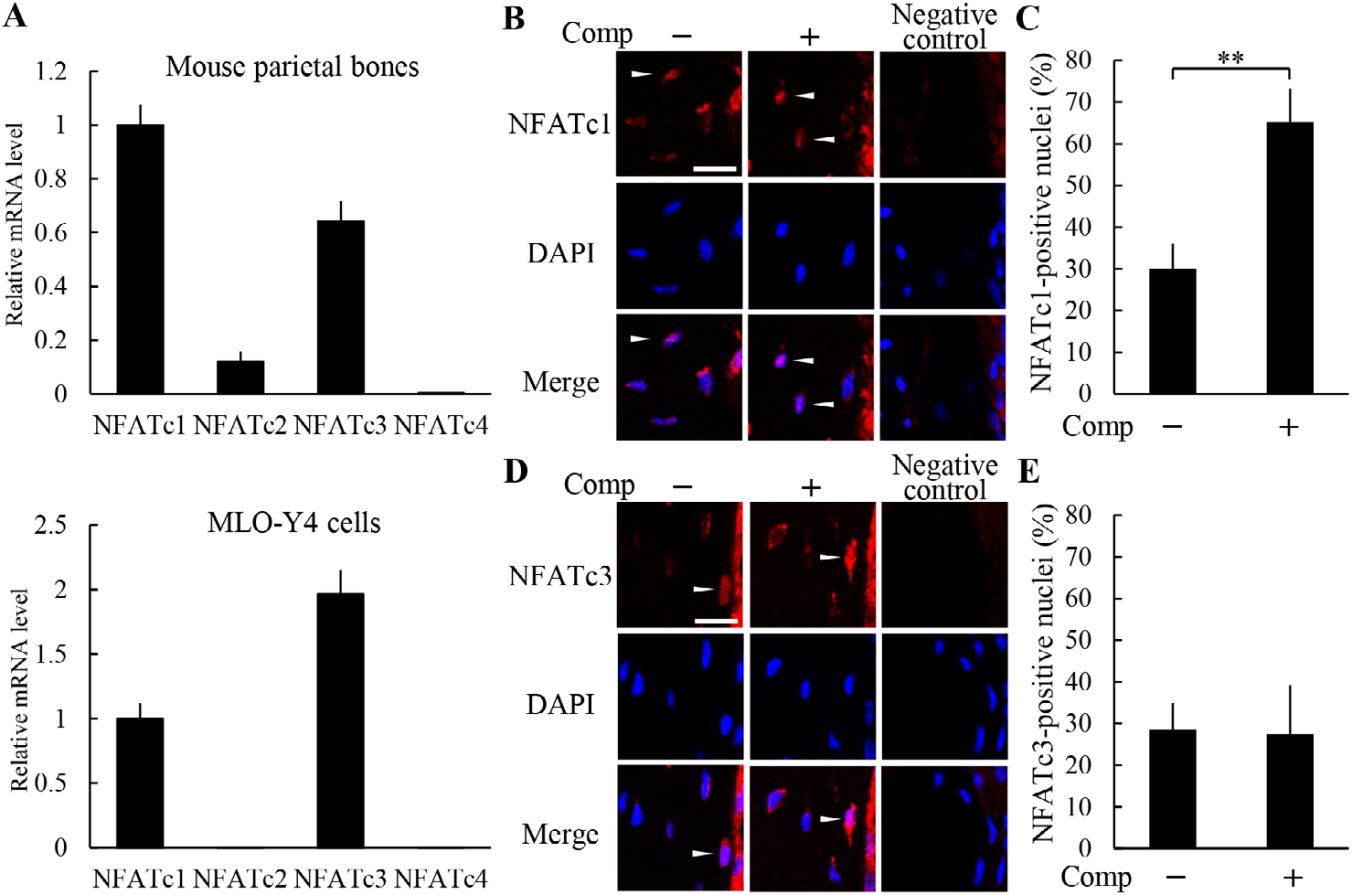
Compressive force induced nuclear translocation of NFATc1 in osteocytes. (*A*) NFATs mRNA expression level in mouse parietal bones and MLO‐Y4 cells was analyzed by real‐time PCR (*n* = 3). Nuclear translocation of NFATc1 (*B,C*) and NFATc3 (*D,E*) in the parietal bones of the nonloaded and loaded groups was analyzed by immunohistochemistry (*B,D*), and the ratio of positive nuclei in osteocytes (*C,E*) was calculated (four animals per group). Arrowheads indicate the nuclear translocation of NFAT in osteocytes. Scale bar = 10 μm. Comp = compressive force loading. **P < 0.01.

We next examined expression of NFATs in the mouse parietal bones and MLO‐Y4 cells. Fig. 5*A* shows NFATc1 and NFATc3 mRNAs were highly expressed in these tissues and cells, whereas expression levels of NFATc2 and NFATc4 mRNAs were quite low. We further analyzed nuclear translocation of NFATc1 and NFATc3 of osteocytes in the compressive force‐loaded parietal bones. The ratio of NFATc1‐positive nuclei was 30.0 ± 6.0% in the nonloaded group, whereas the ratio was significantly increased to 65.2 ± 8.0% in the loaded group (Fig. 5*B*,*C*). The number of NFATc1‐positive nuclei and the total number of nuclei were 3.3 ± 0.5 and 11.0 ± 1.6, and 6.0 ± 0.8 and 9.3 ± 1.3 in the nonloaded and loaded groups, respectively. In contrast, there was no significant difference in the ratio of NFATc3‐positive nuclei between the nonloaded and the loaded groups (Fig. 5*D*,*E*). The number of NFATc3‐positive nuclei and the total number of nuclei were 3.0 ± 0.8 and 10.5 ± 1.3, and 2.8 ± 1.0 and 10.3 ± 1.5 in the nonloaded and loaded groups, respectively. The negative controls of NFATc1 and NFATc3 immunohistochemistries showed no apparent immunopositivity in osteocytes (Fig. 5*B*,*D*).

### The induction of osteocyte apoptosis by compressive force loading is dependent on NFATc1

**Figure 6:**
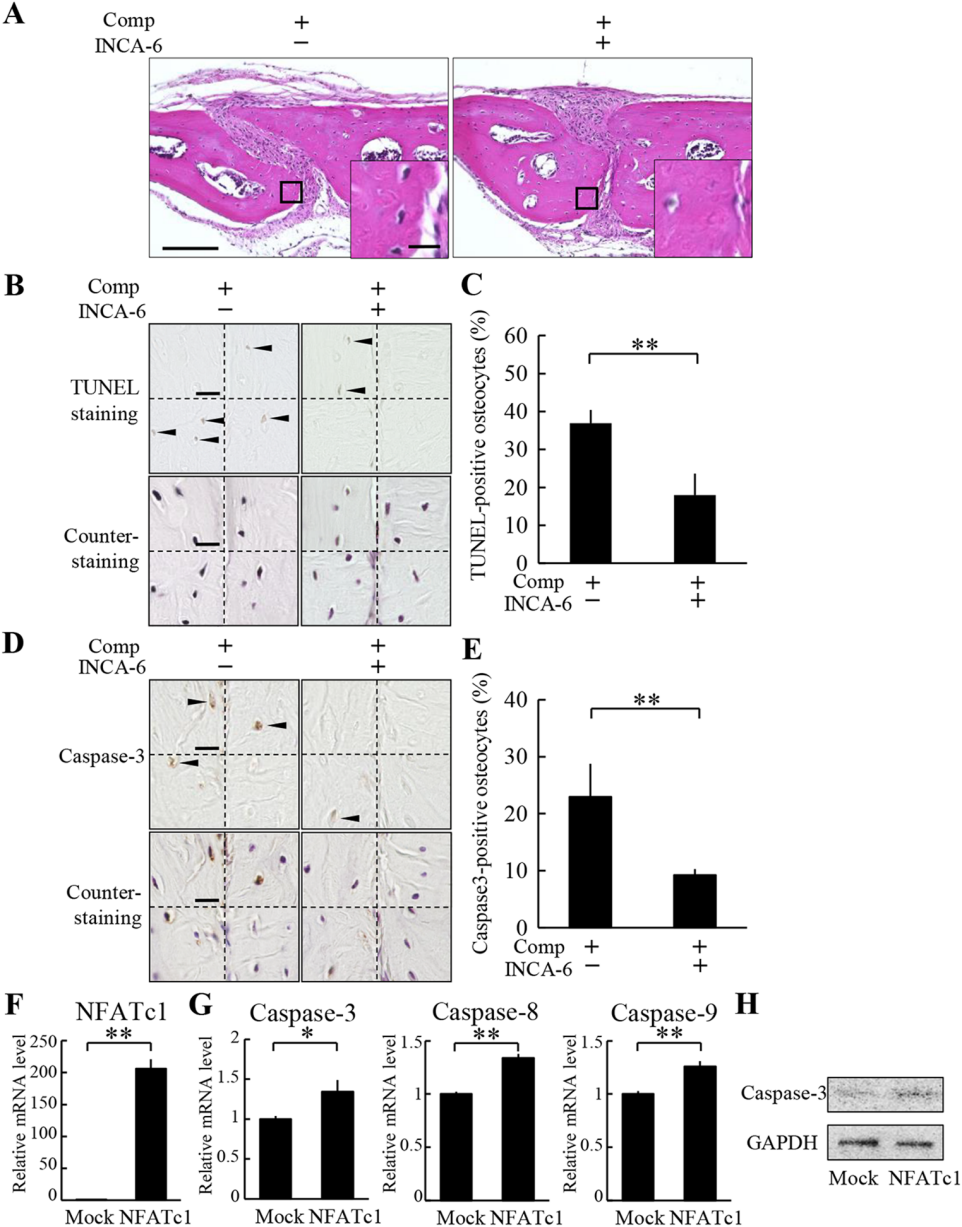
The induction of osteocyte apoptosis by compressive force loading is dependent on NFATc1. (*A*) Histological analysis of the parietal bones at 6 hours after application of compressive force was performed by H&E staining. INCA‐6 was injected s.c. into the parietal bone area prior to the application of compressive force. A magnified picture of the ROI is shown in the lower right corner of each picture. Scale bar = 100 μm, and 10 μm in insets. (*B*) TUNEL staining and subsequent counterstaining with hematoxylin of the parietal bones were performed. All four ROIs in a section in each group are shown. Arrowheads indicate TUNEL‐positive osteocytes. Comp = compressive force loading. Scale bar = 10 μm. (*C*) TUNEL‐positive ratio of osteocytes was calculated (four animals per group). **P < 0.01. (*D*) Caspase‐3 immunohistochemistry and subsequent counterstaining with hematoxylin of the parietal bones were performed. All four ROIs in a section in each group are shown. Arrowheads indicate caspase‐3‐positive osteocytes. Scale bar = 10 μm. (*E*) Caspase‐3‐positive ratio of osteocytes was calculated (four animals per group). **P < 0.01. (*F–H*) MLO‐Y4 cells were infected with a lentiviral vector encoding NFATc1 or an empty vector (mock). The expression of mRNA of NFATc1 (*F*), and caspase‐3, caspase‐8, and caspase‐9 (*G*) in MLO‐Y4 cells were analyzed by real‐time PCR. *P < 0.05, **P < 0.01; *n* = 3. (*H*) Cleaved caspase‐3 expression was analyzed by Western blot.

To examine whether the nuclear translocation of NFATc1 induced by compressive force loading stimulates osteocyte apoptosis; INCA‐6, an inhibitor of nuclear translocation of NFATs, was applied to the parietal bones prior to compressive force loading. No obvious histological changes were observed after INCA‐6 administration in the nonloaded and the loaded groups (Fig. 6*A*). In the compressive force‐loaded group, TUNEL‐positive osteocyte ratio was 37.0 ± 3.5% without INCA‐6 administration, whereas INCA‐6 significantly decreased the ratio to 18.0 ± 5.7% (Fig. 6*B*,*C*). The number of TUNEL‐positive osteocytes and the total number of osteocytes were 3.8 ± 1.3 and 10.0 ± 2.7, and 1.5 ± 0.6 and 8.3 ± 1.3 without and with INCA‐6 administration, respectively. Under the compressive force‐loaded condition, caspase‐3‐positive osteocyte ratio was 22.9 ± 5.8% without INCA‐6 administration, whereas INCA‐6 significantly decreased the ratio to 9.2 ± 1.1% (Fig. 6*D*,*E*). The number of caspase‐3‐positive osteocytes and the total number of osteocytes were 2.3 ± 0.5 and 10.0 ± 1.6, and 1.0 ± 0 and 11.0 ± 1.4 without and with INCA‐6 administration, respectively. To determine a direct effect of NFATc1 on osteocyte apoptosis, we examined expression of apoptosis‐related genes in NFATc1‐overexpressing MLO‐Y4 cells (Fig. 6*F*). Expression levels of caspase‐3, caspase‐8, and caspase‐9 mRNAs in MLO‐Y4 cells were significantly increased by overexpression of NFATc1 (Fig. 6*G*). Moreover, overexpression of NFATc1 upregulated cleaved caspase‐3 protein expression in MLO‐Y4 cells (Fig. 6*H*).

### MENK inhibited nuclear translocation of NFATc1 in the compressive force‐loaded osteocytes

**Figure 7:**
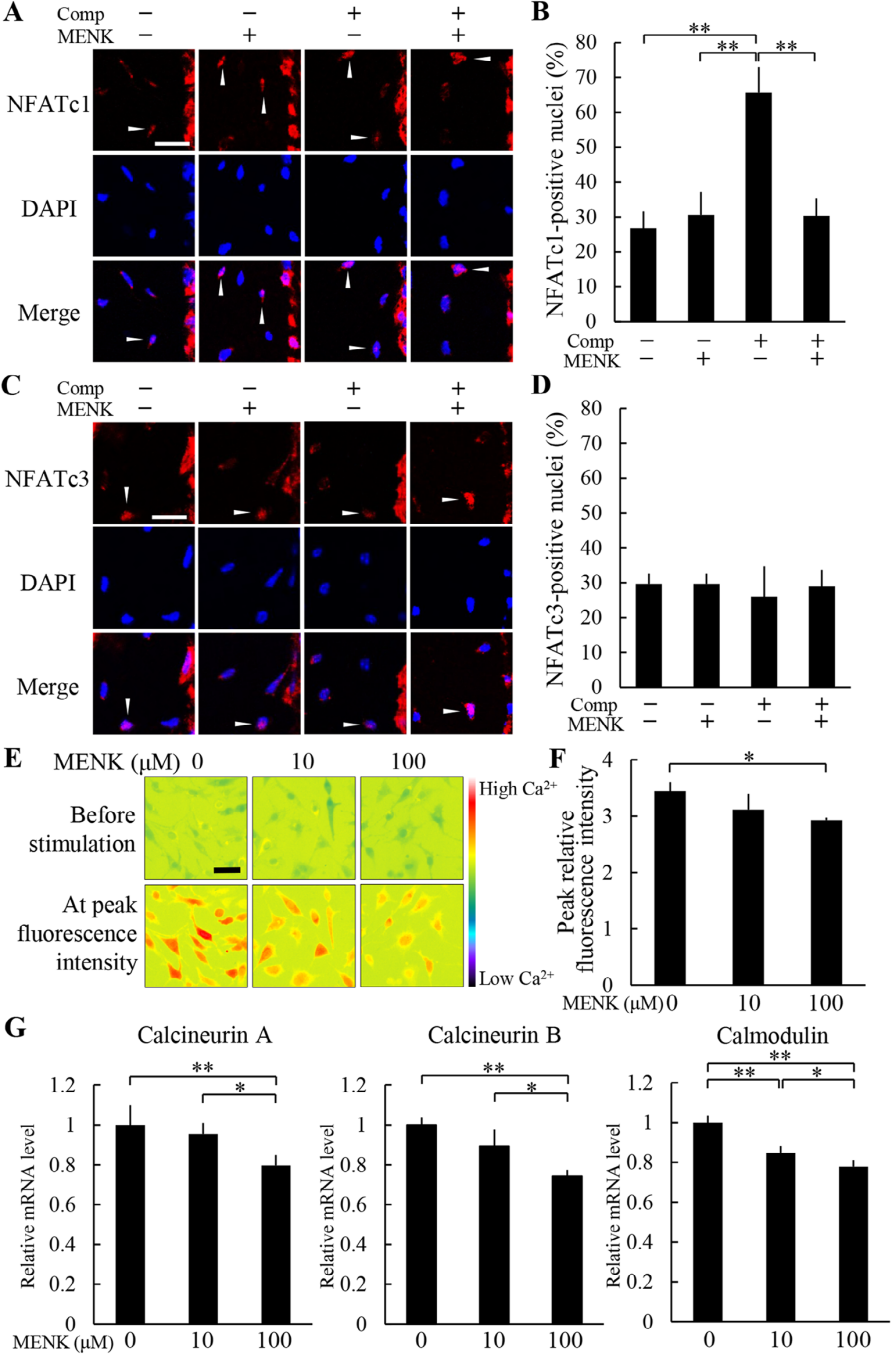
Methionine enkephalin (MENK) inhibited nuclear translocation of NFATc1 in the compressive force‐loaded osteocytes. Nuclear translocation of NFATc1 (*A,B*) and NFATc3 (*C,D*) in the parietal bones at 6 hours after application of compressive force was analyzed by immunohistochemistry (*A,C*), and the ratio of positive nuclei in osteocytes (*B,D*) was calculated (four animals per group.). MENK was injected s.c. into the parietal bone area prior to the application of compressive force. Arrowheads indicate the nuclear translocation of NFAT in osteocytes. Scale bar = 10 μm. **P < 0.01. (*E*) Representative images of Fura‐2 AM‐loaded MLO‐Y4 cells before ionomycin stimulation and at the time of peak fluorescence intensity after ionomycin stimulation. MLO‐Y4 cells were treated with MENK for 24 hours before Ca^2+^ imaging. The color scale represents relative fluorescence intensity. Scale bar = 50 μm. (*F*) Fluorescence intensity before ionomycin treatment was regarded as 1 and the relative increase in Ca^2+^ was measured. Comp = compressive force loading. *P < 0.05; *n* = 3. (*G*) MLO‐Y4 cells were treated with MENK for 24 hours, and the expression levels of calcineurin a, calcineurin B, and calmodulin mRNA in each group at day 2 culture were analyzed by real‐time PCR. *P < 0.05, **P < 0.01; *n* = 4.

Next, we examined whether MENK regulates nuclear translocation of NFATc1 and NFATc3. In the nonloaded groups, ratios of NFATc1‐positive nuclei without and with MENK were 26.7 ± 4.9% and 30.6 ± 6.6%, respectively (Fig. 7*A*,*B*). In the loaded group without MENK, the ratio of NFATc1‐positive nuclei was 65.6 ± 7.4%, a significantly higher ratio than the nonloaded groups (Fig. 7*A*,*B*). In contrast, the ratio in the loaded group with MENK was 30.3 ± 5.0%; it was significantly lower than the loaded group without MENK and was comparable to the nonloaded groups (Fig. 7*A*,*B*). The number of NFATc1‐positive nuclei and the total number of nuclei were 3.0 ± 0 and 11.5 ± 1.9, and 3.3 ± 0.5 and 10.8 ± 1.0 in the nonloaded groups without and with MENK, respectively, and 6.8 ± 1.0 and 10.5 ± 2.7, and 3.5 ± 1.0 and 11.5 ± 2.1 in the loaded groups without and with MENK, respectively. Ratios of NFATc3‐positive nuclei in the nonloaded groups without and with MENK were 29.6 ± 3.0% and 29.6 ± 3.0%, respectively (Fig. 7*C*,*D*). The ratios in the loaded groups without and with MENK were 26.0 ± 8.8% and 28.9 ± 4.7%, respectively (Fig. 7*C*,*D*). There was no significant difference between any of the groups. The number of NFATc3‐positive nuclei and the total number of nuclei were 3.5 ± 0.6 and 11.8 ± 1.0, 3.5 ± 0.6, and 11.8 ± 1.0 in the nonloaded groups without and with MENK, respectively, 3.0 ± 1.4 and 11.3 ± 1.7, and 3.0 ± 0.8 and 10.3 ± 1.3 in the loaded groups without and with MENK, respectively. These data indicated that MENK inhibited the nuclear translocation of NFATc1 in osteocytes induced by compressive force loading.

To further investigate the mechanisms by which MENK regulates nuclear translocation of NFATc1, we focused on Ca^2+^signaling. Real‐time Ca^2+^ imaging using the Ca^2+^ indicator dye Fura‐2 AM showed that a Ca^2+^ ionophore ionomycin elevated the fluorescence intensity of intercellular Ca^2+^ in MLO‐Y4 cells without MENK treatment (Fig. 7*E*,*F*). The elevation of fluorescence intensity by ionomycin was decreased by MENK treatment in a dose‐dependent manner; higher concentration of MENK showed significant reduction of the relative increase in fluorescence compared with the MENK‐nontreated control (Fig. 7*E*,*F*). Moreover, the expression of Ca^2+^ signaling mediators, calcineurin A, calcineurin B, and calmodulin in MLO‐Y4 cells were assessed. Expression levels of these mRNAs were significantly decreased by MENK treatment in a dose‐dependent manner (Fig. 7*G*).

## Discussion

It is apparent that neuropeptides play important roles in bone metabolism, and the identification of neuropeptides that promote bone remodeling is a critical issue in bone biology.^(^ 14, 15, 16 ^)^ It is generally thought that neuropeptides are produced by nerve cells, but recent studies indicate that bone cells are also producers of neuropeptides. For instance, neuropeptide Y is produced by osteocytes and facilitates bone remodeling.^(^ ^37^ ^)^ In the present study, we demonstrated, for the first time, that osteocytes produce an endogenous opioid MENK in the mouse parietal bones. In addition, we found the expression of DOR, a major receptor of MENK, in osteocytes. Osteocytes reside in lacunae within the mineralized bone matrix and have their dendritic processes through tiny tunnels called canaliculi, forming the osteocyte lacunocanalicular network via gap junctions.^(^ 2, 3, 38, 39 ^)^ These findings suggest that MENK functions in an autocrine and/or paracrine manner in the osteocyte lacunocanalicular network.

Osteocytes are key players of mechanotransduction in bones; that is, they convert external mechanical stress into biochemical signaling.^(^ 5, 38 ^)^ CTGF is known as a mechanoresponsive factor; its expression is upregulated by mechanical stress.^(^ 12, 13 ^)^ It binds to candidate receptors, such as integrin, lipoprotein receptor‐related protein‐1, and tyrosine kinase receptor A; its signaling facilitates various biological processes, including proliferation, differentiation, and substrate production.^(^ 40, 41, 42, 43 ^)^ We previously reported that increased expression of CTGF in osteocytes by compressive force induces osteocyte apoptosis in vivo and in vitro.^(^ 12, 13 ^)^ In the present study, we loaded compressive force to the mouse parietal bones, resulting in a reduction of MENK expression in osteocytes. A neutralizing CTGF antibody inhibited the compressive force‐induced reduction of MENK. Furthermore, we showed that an increase in osteocyte apoptosis in the compressive force‐loaded parietal bones was inhibited by MENK administration. These results suggest that osteocyte apoptosis in response to compressive force is induced through suppression of MENK expression by CTGF signaling. Osteocyte apoptosis is regulated positively by glucocorticoids and oxidative stress‐related factors and negatively regulated by PTH, estrogen, and androgen.^(^ ^44^ ^)^ In addition to these factors, neuropeptides, including MENK, can be considered novel regulators of osteocyte apoptosis according to the present study. Aging, loss of mechanical stress under a condition of weightlessness, and glucocorticoid‐induced bone disease cause excessive osteocyte apoptosis and subsequent bone loss.^(^ 45, 46, 47 ^)^ Our finding about the inhibitory effect of MENK on osteocyte apoptosis may contribute to the development of treatments for abnormal bone loss caused by osteocyte apoptosis.

Because NFAT has a close relationship with apoptosis in various types of cells, we examined the role of NFAT in compressive force‐induced osteocyte apoptosis. Our data showed that NFATc1 and NFATc3 mRNA were highly expressed in bone tissues and osteocytes. Nuclear translocation of NFATc1 in the osteocytes in the parietal bones was enhanced by compressive force, whereas that of NFATc3 was not. These results indicated that translocation of NFATc1, among NFATs, into nuclei was increased in response to the compressive force in osteocytes. INCA‐6, which inhibits NFAT translocation into nuclei, suppressed the increase in osteocyte apoptosis in the compressive force‐loaded parietal bones. Moreover, overexpression of NFATc1 induced expression of apoptosis‐related genes and cleaved caspase‐3 protein in MLO‐Y4 cells. Our findings suggest that osteocyte apoptosis induced by compressive force is advanced through induction of NFATc1 nuclear translocation.

Furthermore, we analyzed the relationship between MENK and NFATc1 in the molecular regulation of osteocytes apoptosis. Our data showed that MENK administration reduced nuclear translocation of NFATc1 in osteocytes in vivo, suggesting that the decreased MENK expression promotes nuclear translocation of NFATc1 and following osteocyte apoptosis during osteocyte response to compressive force. It is known that the Ca^2+^ signaling pathway is a major mechanism of nuclear translocation of NFAT. That is, Ca^2+^ influx into cytoplasm leads to calmodulin‐dependent activation of calcineurin, followed by NFAT dephosphorylation and nuclear translocation.^(^ ^48^ ^)^ Importantly, the Ca^2+^/calcineurin/NFAT pathway promotes apoptosis in various cells such as muscle cells, megakaryocytes, and mesangial cells.^(^ 33, 49, 50 ^)^ In the present study, we found that MENK suppressed Ca^2+^ influx and expression of calcineurin and calmodulin in osteocytes, indicating that MENK is a negative regulator of Ca^2+^ signaling. Taken together, MENK would negatively regulate osteocyte apoptosis through the suppression of the Ca^2+^/calcineurin/NFATc1 pathway.

Ca^2+^ influx is an initial biochemical event of mechanical stress‐loaded osteocytes; it stimulates osteocyte functions, such as gene expression and intercellular communication.^(^ ^51^ ^)^ Therefore, inhibition of Ca^2+^ influx by MENK may be an important mechanism to regulate mechanical stress‐induced bone remodeling by regulating not only apoptosis, but other functions of osteocytes.

## Conclusions

The present study revealed that osteocytes expressed MENK, whereas the MENK expression was suppressed by compressive force via CTGF signaling. Moreover, MENK inhibited compressive force‐induced osteocyte apoptosis by downregulating nuclear translocation of NFATc1. MENK is considered to regulate nuclear translocation of NFATc1 by suppressing Ca^2+^ signaling in osteocytes. These results suggest that osteocyte‐produced MENK is a competent neuropeptide that regulates bone remodeling driven by mechanical stress. Our findings about novel roles of MENK in osteocytes not only contribute to understanding the mechanisms of bone remodeling regulated by neuropeptides, but are also possibly applicable to the development of treatments for pathological bone loss.

## Disclosures

All authors state that they have no conflicts of interest.
